# Endemic coronavirus infection is associated with SARS-CoV-2 Fc receptor-binding antibodies

**DOI:** 10.1128/jvi.00550-25

**Published:** 2025-05-19

**Authors:** David J. Bean, Yan Mei Liang, Frida Avila, Xianbao He, Archana Asundi, Manish Sagar

**Affiliations:** 1 Department of Virology, Immunology and Microbiology, Boston University Chobanian and Avedisian School of Medicine 12259, Boston, Massachusetts, USA; 2 Department of Medicine, Boston University Chobanian and Avedisian School of Medicine 12259, Boston, Massachusetts, USA; Loyola University Chicago - Health Sciences Campus, Maywood, Illinois, USA

**Keywords:** COVID-19, SARS-CoV-2, endemic coronavirus, antibody function, Fc receptors, adaptive immunity

## Abstract

Recent documented infection with an endemic coronavirus (eCoV) is associated with less severe coronavirus disease 2019 (COVID-19), yet the immune mechanism behind this protection has not been fully explored. We measured both antibody and T-cell responses against severe acute respiratory syndrome coronavirus 2 (SARS-CoV-2) in SARS-CoV-2-naïve individuals, classified into two groups: those with or without presumed recent eCoV infections. There was no difference in neutralizing antibodies and T-cell responses against SARS-CoV-2 antigens between the two groups. SARS-CoV-2-naïve individuals with recent presumed eCoV infection, however, had higher and significantly correlated levels of Fc receptor (FcR)-binding antibodies against eCoV spikes (S) and SARS-CoV-2 S2. Recent eCoV infection boosts cross-reactive antibodies that can mediate Fc effector functions, and this may play a role in the observed heterotypic immune protection against severe COVID-19.

**IMPORTANCE:**

With the recent emergence of SARS-CoV-2 and other pathogenic coronaviruses, it is important to understand how the immune system may protect against disease from future coronavirus outbreaks. We investigated the adaptive immune responses elicited from a “common cold” eCoV and measured the cross-reactivity against SARS-CoV-2 in individuals classified as having or not having a recent eCoV infection. Although both groups had similar cross-reactive T-cell and neutralizing antibody responses, individuals with a recent eCoV infection had higher antibody levels capable of Fc receptor binding. Antibodies with enhanced Fc receptor binding could mediate the killing of virally infected cells through mechanisms such as antibody-dependent cellular cytotoxicity, which may reduce the severity of COVID-19. Antibodies capable of mediating Fc effector functions may be critical for therapies and vaccines against future pathogenic coronavirus outbreaks.

## INTRODUCTION

Prior to the emergence of severe acute respiratory syndrome coronavirus 2 (SARS-CoV-2) and coronavirus disease 2019 (COVID-19), studies suggested that humans are infected with an endemic coronavirus (eCoV), such as human coronavirus (HCoV)-229E, HCoV-HKU1, HCoV-NL63, and HCoV-OC43, from about every 18 months to 2 years (1, 2). It has been hypothesized that cross-reactive immune responses generated from one coronavirus (CoV) may protect against infection or ameliorate disease severity from a heterologous CoV (3, 4). In a large retrospective cohort study, we observed that individuals with a documented recent eCoV infection had less severe COVID-19, although there was no difference in SARS-CoV-2 incidence (5). The immune mechanism behind this observed protection has not been definitively elucidated. Some, but not all, studies suggest that the pre-existing humoral and cellular immunity may decrease SARS-CoV-2 incidence and COVID-19 severity (6–8). Few studies, however, have directly looked at how the cross-reactive immune response changes after a recent eCoV infection.

Recent eCoV infections are difficult to diagnose because the disease is mild, and the etiology for a “common cold” is usually not investigated if one presents for medical care (9). Many studies define recent eCoV infections by detecting viral RNA through polymerase chain reaction (PCR)-based methods or changes in eCoV-directed antibody levels (1, 10). Multiplex comprehensive respiratory panel (CRP)-PCR tests detect active eCoV infections (11), but they are not widely used, and most individuals do not present for medical care when they have the common cold due to an eCoV (10). Detecting changes in antibody levels requires longitudinal sampling, and the number of participants and longevity of the study hinder these types of studies. Inability to identify individuals with recent eCoV infections with confidence makes it difficult to assess the effect on SARS-CoV-2 immunity.

In this study, we used a combination of PCR-documented eCoV infections with eCoV nucleocapsid antibody titer data to classify individuals with presumed recent eCoV infections. We compared cross-reactive immune responses to various SARS-CoV-2 antigens between individuals with or without a presumed recent eCoV infection. We found that eCoV spike-specific antibodies are boosted after a recent eCoV infection and are likely mediating the cross-reactivity against SARS-CoV-2 spike. These cross-reactive antibodies were not associated with higher SARS-CoV-2 neutralization, but they were capable of binding to the Fc receptor (FcR), FcγRIIIa, and potentially mediating enhanced Fc effector functions. Our results suggest that a recent infection with an eCoV boosts the Fc effector function potential of CoV-specific antibodies and offers an additional possible mechanism for the protection against severe COVID-19.

## RESULTS

### Plasma IgG levels indicate recent CoV infections

**Fig 1:**
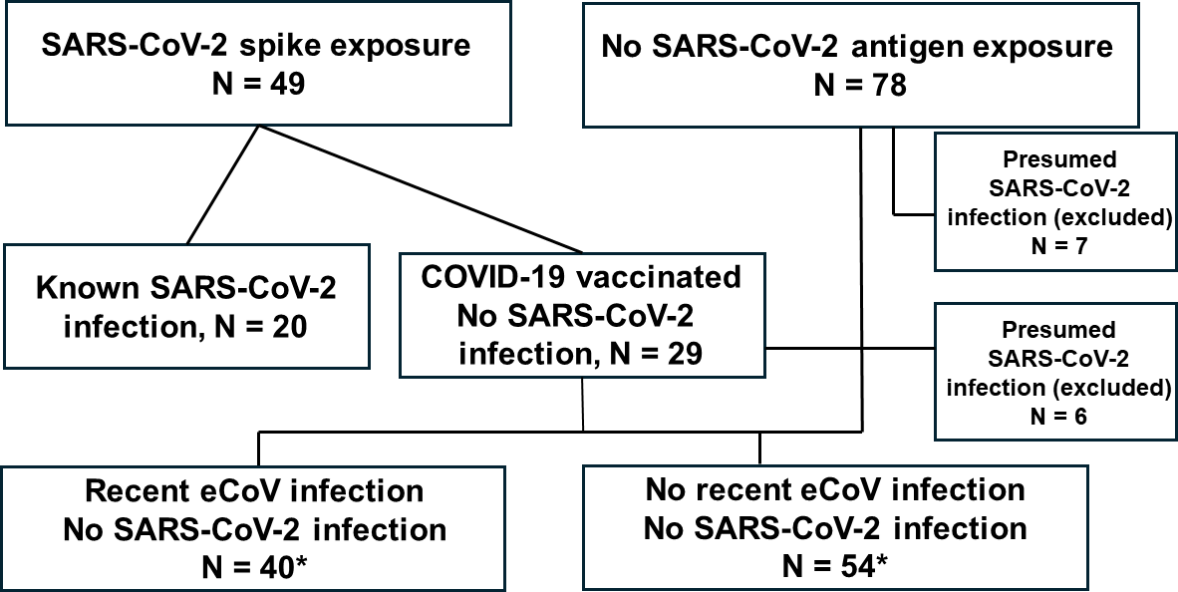
Diagram of different groups in this study. The SARS-CoV-2 spike-exposed group consisted of individuals with documented SARS-CoV-2 infection and those with COVID-19 vaccination but no known SARS-CoV-2 infection. The no SARS-CoV-2 infection group with and without recent eCoV infection consisted of individuals with no known SARS-CoV-2 antigen exposure and prior COVID-19 vaccine only. The asterisk highlights that individuals with prior COVID-19 vaccination were not included when analyzing SARS-CoV-2 spike responses in the group with (*n* = 13) and without (*n* = 10) recent eCoV infection.

**Fig 2:**
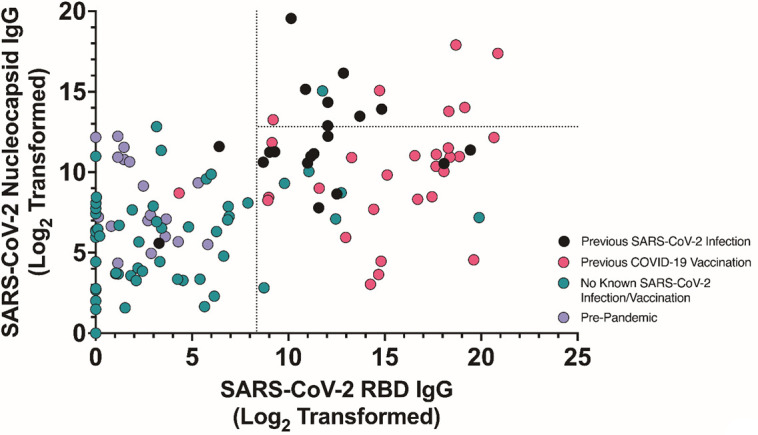
Classification of presumed SARS-CoV-2 infections based on antibody levels. IgG antibody levels against SARS-CoV-2 RBD and nucleocapsid protein were measured in individuals with a previous SARS-CoV-2 infection (black), previous COVID-19 vaccination with no documented history of SARS-CoV-2 infections (pink), no known SARS-CoV-2 infection or COVID-19 vaccination (green), or pre-pandemic samples collected before March 2020 (purple). The dotted lines specify cutoffs that were established to classify individuals with presumed undiagnosed SARS-CoV-2 infection.

The primary aim of this single-center study was to compare *ex vivo* SARS-CoV-2 immune responses among individuals classified based on recent eCoV infection status. We prospectively collected samples from individuals with confirmed previous SARS-CoV-2 spike exposure (known SARS-CoV-2 infection [*n* = 20] or prior COVID-19 vaccination but no documented SARS-CoV-2 infection [*n* = 29]) or no known prior SARS-CoV-2 antigen history (no SARS-CoV-2 infection or COVID-19 vaccination [*n* = 78]) (Fig. 1; Table S1). We sought to identify undocumented SARS-CoV-2 infections among those with prior COVID-19 vaccination only or no known SARS-CoV-2 exposure. Eighteen individuals were sampled prior to the start of the COVID-19 pandemic in the United States, and thus, these individuals were SARS-CoV-2 naïve. The remaining people (*n* = 89), however, possibly could have had prior SARS-CoV-2 infection because samples were collected after March 2020. We used the previous methodology described by our group and others to identify individuals who may have had undocumented asymptomatic SARS-CoV-2 infection (4, 12). Briefly, we measured plasma IgG levels against SARS-CoV-2 receptor-binding domain (RBD) and SARS-CoV-2 nucleocapsid by enzyme-linked immunosorbent assay (ELISA). ELISA results on samples collected prior to the pandemic, those with known SARS-CoV-2 infection, and those with prior COVID-19 vaccination were used to set cutoffs for the SARS-CoV-2 RBD and nucleocapsid IgG levels that would differentiate individuals with possible asymptomatic undocumented SARS-CoV-2 infections (Fig. 2). As expected, most of the individuals with either a previous SARS-CoV-2 infection (18 out of 20, 90%) or COVID-19 vaccination (28 out of 29, 97%) had elevated anti-SARS-CoV-2 RBD IgG titers. Furthermore, all the pre-pandemic samples (18 out of 18, 100%) were below the set cutoff for SARS-CoV-2 antigen exposure. In summary, the overall classification accuracy based on our assigned cutoffs was 87% (95% confidence interval [CI] 76%–94%), which is within the range of previous methodologies (73%–91%) (Table S2) (4). Seven individuals classified in the no SARS-CoV-2 antigen exposure group had IgG levels against SARS-CoV-2 RBD above the established cutoff, indicative of a potential previous SARS-CoV-2 infection, and these seven were excluded from all further analysis. Furthermore, anti-SARS-CoV-2 nucleocapsid IgG levels above the established cutoff indicated that six individuals with a prior COVID-19 vaccination might have had a previous SARS-CoV-2 infection. These 6 were also excluded from groups deemed to have no SARS-CoV-2 infection, and thus, we had 94 individuals deemed as not having previous SARS-CoV-2 infection (Fig. 1).

This work primarily aimed to understand anti-SARS-CoV-2 immune responses among those with and without recent eCoV exposure in the absence of SARS-CoV-2 infection. Thus, we examined the 94 individuals without a previous documented or suspected SARS-CoV-2 infection to identify individuals with a recent eCoV infection. Longitudinal sampling in prior studies suggests a reinfection with an eCoV occurs on average every 1–3 years (1, 2). Furthermore, the duration between peak antibody responses after a presumed eCoV infection suggests that a subsequent infection with a heterologous eCoV occurs around every 1.5 years. Based on this time frame and the Boston Medical Center (BMC) electronic medical record (EMR), we identified 22 individuals who had documented eCoV RNA (11 HCoV-OC43, 6 HCoV-NL63, 4 HCoV-229E, and 1 HCoV-HKU1) on a prior multiplex CRP PCR test less than 550 days prior to the sample collected for this study. These individuals were classified as having a recent eCoV infection. To identify other individuals with presumed recent eCoV infections in the no prior SARS-CoV-2 infection group, we measured plasma IgG antibody levels against all four human eCoV nucleocapsid proteins, similar to previously used methods (13). Nucleocapsid antibody titers against the two alpha (HCoV-229E and HCoV-NL63, Pearson *ρ* = 0.920, *P* < 0.0001; Fig. S1A) and the two beta (HCoV-OC43 and HCoV-HKU1, Pearson *ρ* = 0.478, *P* < 0.0001; Fig. S1B) eCoVs showed significant correlations. On the other hand, there was no correlation between alpha and beta nucleocapsid (Fig. S1C). Thus, we generated a composite alpha and beta eCoV anti-nucleocapsid IgG metric. The top 25% of individuals (*n* = 18) with the highest nucleocapsid antibody levels (either anti-alpha or beta eCoV) were classified as having a recent eCoV but no SARS-CoV-2 infection. The rest (*n* = 54) were classified as not having a recent eCoV or SARS-CoV-2 infection. Other than a clinically small but statistically significant age difference, the two groups classified based on recent eCoV infection status had no meaningful demographic differences (Table 1). In all subsequent analyses of spike-directed immune responses, the individuals with prior COVID-19 vaccination but no SARS-CoV-2 infection were not included in the two groups with different recent eCoV infection history.

**TABLE 1:** Comparison of demographics of the individuals with or without a presumed recent eCoV exposure and no SARS-CoV-2 infection^*k*^

	Presumed or documented recent eCoV infection (*n* = 40)	No presumed recent eCoV infection (*n* = 54)	*P* value^*a*^
Age (years), median (interquartile range)	63 (50–70)	56 (45–63)	0.0470^*b*^
Male	19 (48)	28 (52)	0.6765
Race/ethnicity^*c*^			0.8105
Black	18 (45)	29 (54)	
White	16 (40)	18 (33)	
Hispanic/Latino	5 (13)	5 (9)	
Other/missing	1 (3)	2 (4)	
Diabetes mellitus	14 (35)	18 (33)	0.8661
Heart disease^*d*^	15 (38)	11 (20)	0.0664
Lung disease^*e*^	17 (43)	15 (28)	0.1364
CKD^*f*^	3 (8)	6 (11)	0.7283^*g*^
HIV^*h*^	8 (20)	18 (33)	0.1530
Cancer	1 (3)	2 (4)	>0.9999^*g*^
Number of comorbidities^*i*^			0.4396
0	7 (18)	8 (15)	
1	14 (35)	26 (48)	
≥2	19 (48)	20 (37)	
Pre-pandemic^*j*^	4 (10)	14 (26)	0.0656^*g*^
COVID-19 vaccine	13 (33)	10 (19)	0.1190

^*a*^ χ test unless otherwise indicated.

^*b*^ Mann-Whitney test.

^*c*^ As specified in the EMR, an individual may be in more than one category.

^*d*^ Heart disease includes coronary artery disease and congestive heart failure.

^*e*^ Lung disease includes chronic obstructive pulmonary disease and asthma.

^*f*^ Chronic kidney disease.

^*g*^ Fisher’s exact test.

^*h*^ Human immunodeficiency virus.

^*i*^ Number of comorbidities accounts for diabetes mellitus, heart disease, lung disease, chronic kidney disease, HIV, and cancer.

^*j*^ Samples collected before March 2020.

^*k*^ Data show the number and percent unless otherwise indicated.

### Recent eCoV infection is associated with increased SARS-CoV-2 S2-specific FcγRIIIa-binding antibody responses

**Fig 3:**
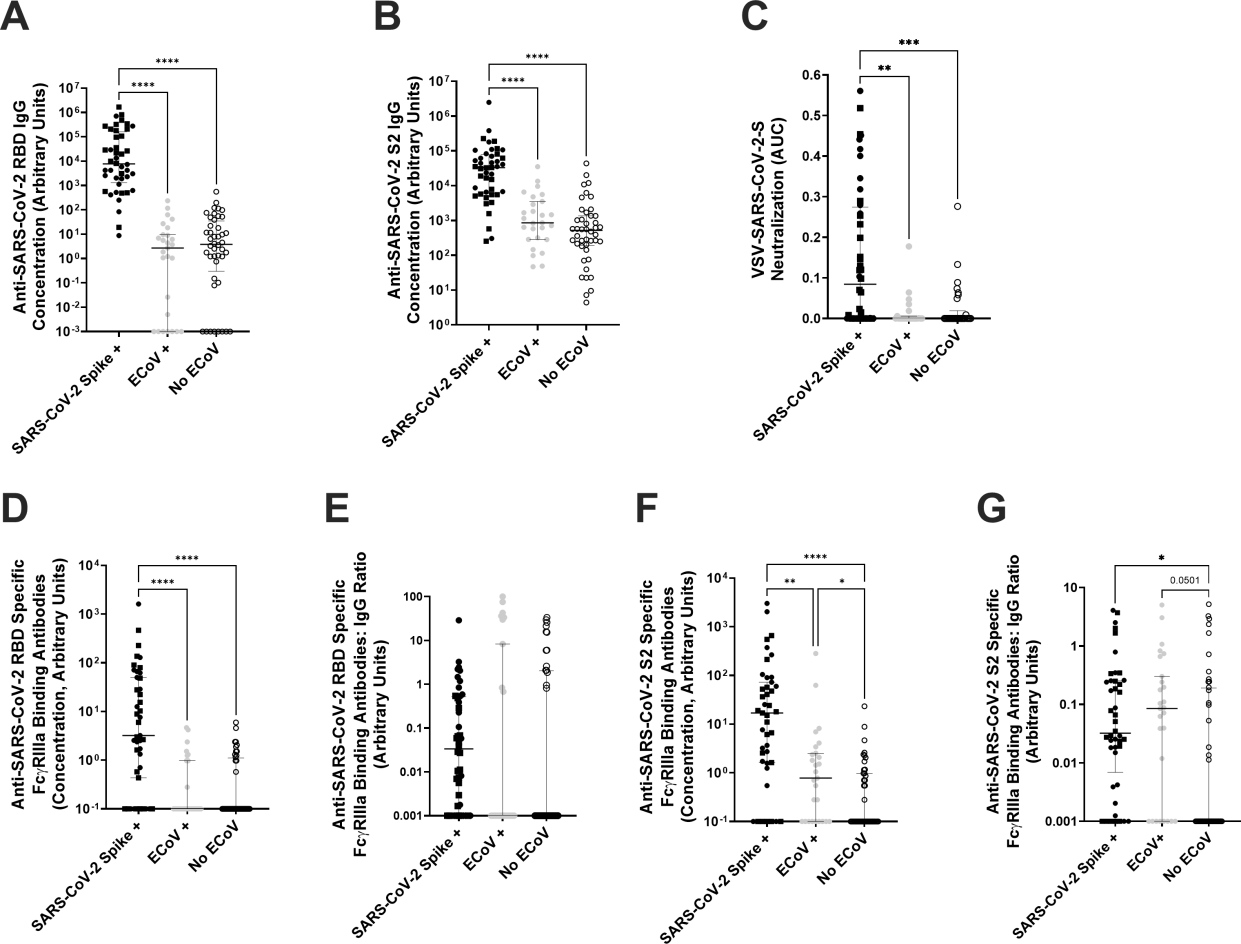
Humoral immune responses to SARS-CoV-2 spike antigens. Plasma antibody responses in those with documented SARS-CoV-2 spike exposure (previous SARS-CoV-2 infection [black circle] or prior COVID-19 vaccination [black square]), presumed or documented recent eCoV infection (gray circle), or without a presumed recent eCoV infection (white circle). (**A and B**) IgG antibody levels against SARS-CoV-2 RBD (**A**) or SARS-CoV-2 spike S2 subunit (**B**). (**C**) Neutralization responses against a VSV-ΔG pseudovirus expressing the SARS-CoV-2-Wuhan spike protein. (**D–G**) Titer of SARS-CoV-2 RBD (**D**) or SARS-CoV-2 S2 (**F**)-specific antibodies binding to the Fc receptor, FcγRIIIa, or the ratio of those FcR-binding antibodies to the SARS-CoV-2 RBD (**E**) or SARS-CoV-2 S2 (**G**) IgG levels in panels **A and B**. The dark horizontal lines in each scatter dot plot denote the median and interquartile range. Statistical analyses were performed using either the Kruskal-Wallis test with the Dunn multiple comparison test or the Mann-Whitney *U* test. *P* values less than 0.1 are displayed. *<0.05, **<0.01, ***<0.001, and ****<0.0001.

We examined cross-reactive humoral immunity after a recent eCoV infection because antibody responses are the best correlate of protection against SARS-CoV-2 infection and severe COVID-19 (14, 15). There was no significant difference in anti-SARS-CoV-2 RBD IgG (Fig. 3A) and anti-SARS-CoV-2 S2 (Fig. 3B) IgG antibodies in those with and without a recent eCoV infection. As expected, anti-SARS-CoV-2 spike antibodies were significantly higher in the individuals with prior SARS-CoV-2 spike exposure (Fig. 3A and B). Neutralization responses against vesicular stomatitis virus (VSV) pseudotyped with SARS-CoV-2 spike (Wuhan variant) were higher in those with previous SARS-CoV-2 spike exposure, but there was no difference in neutralization between individuals with or without a presumed recent eCoV infection (Fig. 3C).

Another potential functional role of these anti-SARS-CoV-2 antibodies is through FcR binding, which contributes to the clearance of virally infected cells through mechanisms such as antibody-dependent cellular cytotoxicity (ADCC) or antibody-dependent cellular phagocytosis (ADCP) (16). We used a previously validated ELISA method to measure SARS-CoV-2 specific FcγRIIIa (CD16a)-binding antibodies, which is a predictor for natural killer (NK) cell-mediated antibody effector function (17). As expected, those individuals with prior SARS-CoV-2 spike exposure had higher levels of anti-SARS-CoV-2 RBD and S2 antibodies binding to FcγRIIIa (Fig. 3D through G). These elevated SARS-CoV-2-specific FcR binding responses were not different between those individuals with either a previous SARS-CoV-2 infection or COVID-19 vaccination (Fig. S2). Individuals with or without a presumed recent eCoV infection had similar anti-SARS-CoV-2 RBD FcγRIIIa-binding antibodies and FcγRIIIa-binding antibodies-to-IgG ratio (Fig. 3D and E). However, anti-SARS-CoV-2 S2 FcγRIIIa-binding antibodies were 7.8-fold higher (*P* = 0.0234), and the FcγRIIIa-binding antibodies-to-IgG ratio was 85-fold higher (*P* = 0.0501) in the individuals classified as having a recent eCoV infection as compared to those not having a recent eCoV exposure (Fig. 3F and G).

**Fig 4:**
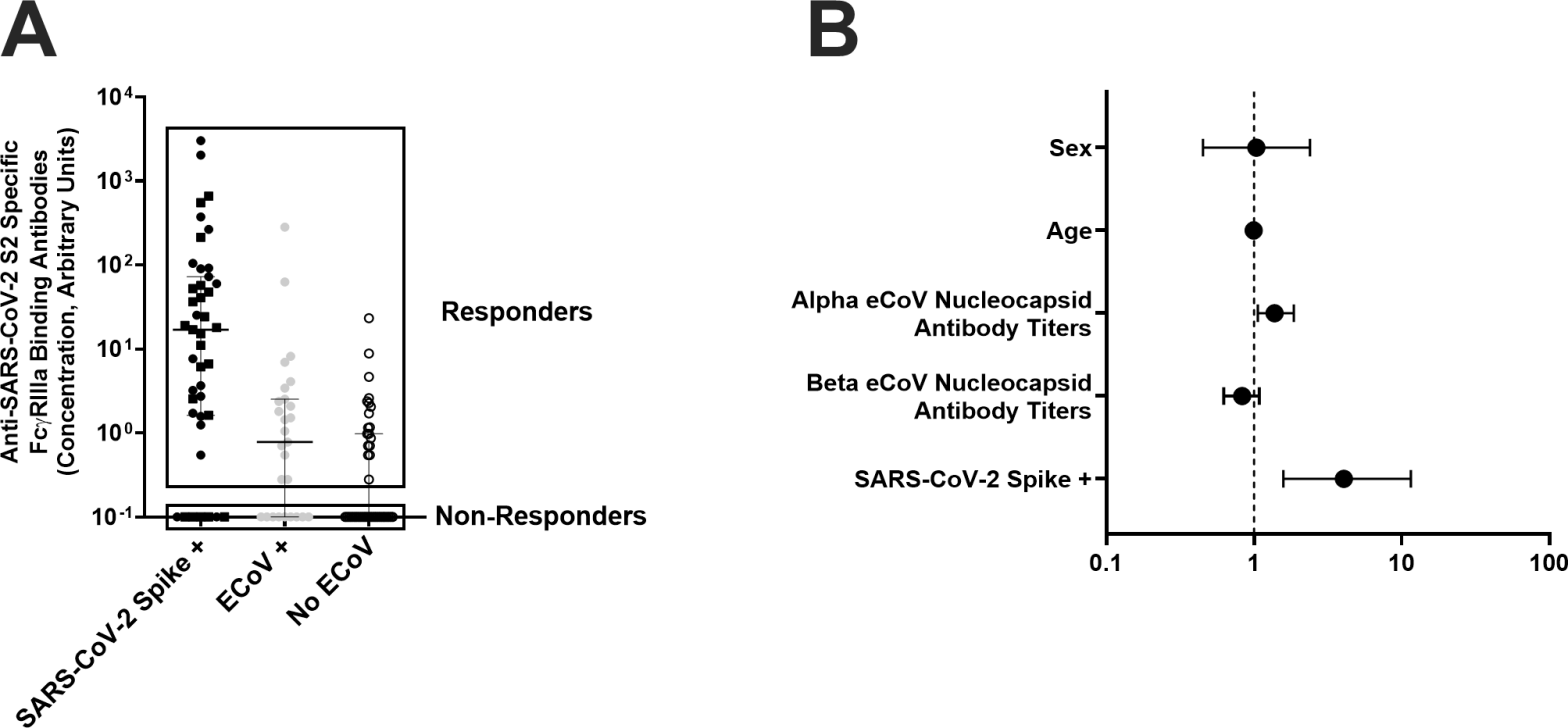
Higher alpha eCoV nucleocapsid antibody responses predict positive responses in the SARS-CoV-2 S2-specific antibody FcR-binding assay. (**A**) Individuals were separated into two groups based on positive (responders) or absent (non-responders) FcγRIIIa SARS-CoV-2 S2-specific antibody binding. These responder and non-responder classifications were used in a multivariate logistic regression model. These data are the same as those in Fig. 2F. (**B**) The odds ratio of variables used in the multivariate logistic regression model to predict positive SARS-CoV-2 S2 antibody FcR binding responses. The error bars represent the 95% CIs.

In the FcγRIIIa-binding assay, individuals could be separated into responders and non-responders based on FcγRIIIa binding activity above or below a designated cutoff (Fig. 4A). We used a multiple logistic regression model to identify characteristics associated with SARS-CoV-2 S2-specific FcγRIIIa antibody binding. As expected, previous SARS-CoV-2 spike exposure was associated with 4.055-fold higher odds (95% CI 1.581–11.55, *P* = 0.0053) of having a SARS-CoV-2 S2 FcγRIIIa-binding antibody response. There were 1.379-fold higher odds (95% CI 1.060–1.859, *P* = 0.0249) of having a SARS-CoV-2 S2 FcγRIIIa-binding antibody response for every 2-fold increase in anti-alpha eCoV nucleocapsid antibody levels (Fig. 4B). Levels of anti-beta eCoV nucleocapsid antibodies, age, gender, and other demographic characteristics were not associated with detectable SARS-CoV-2 S2 FcγRIIIa-binding antibody.

### SARS-CoV-2 S2 FcγRIIIa-binding antibody responses are correlated with eCoV spike-specific FcγRIIIa-binding antibody responses

**Fig 5:**
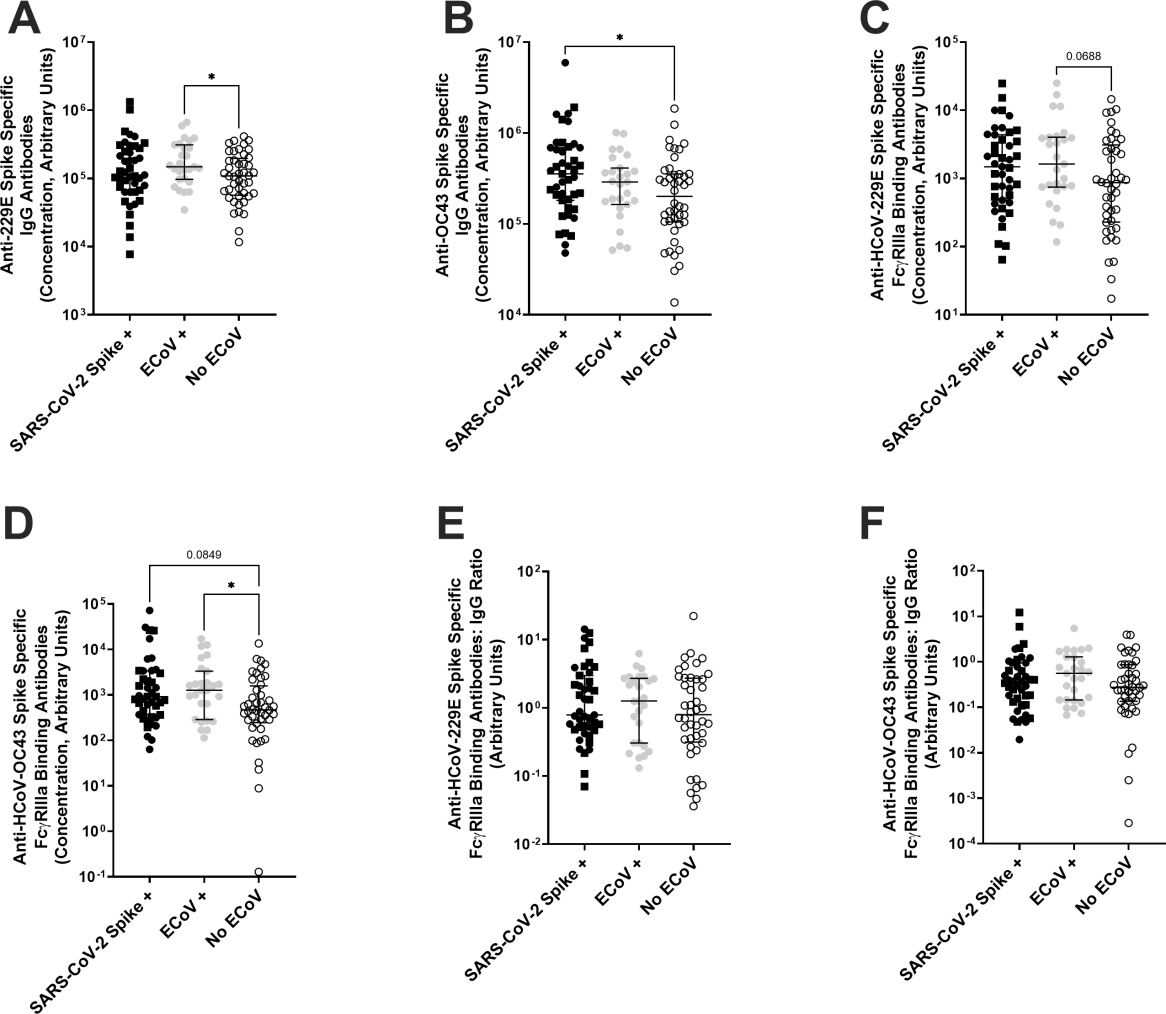
ECoV-specific antibody FcR binding responses increased after a recent eCoV infection. Plasma antibody responses were measured in individuals with either a previous SARS-CoV-2 spike exposure (previous SARS-CoV-2 infection [black circle] and previous COVID-19 vaccination [black square]), presumed or documented recent eCoV infection (gray circle), or without a presumed recent eCoV infection (white circle). (**A and B**) IgG antibody responses against HCoV-229E (**A**) or HCoV-OC43 (**B**) spikes. (**C and D**) Level of HCoV-229E (**C**) or HCoV-OC43 (**D**) spike-specific antibodies binding to the Fc receptor, FcγRIIIa. (**E and F**) Ratio of Fc receptor antibody binding (**C and D**) to antigen-specific IgG (**A and B**) in HCoV-229E (**E**) or HCoV-OC43 (**F**) spike-specific antibody responses. The dark horizontal lines in each plot denote the median and interquartile range. Statistical analyses were performed using either the Kruskal-Wallis test with the Dunn multiple comparison test or the Mann-Whitney *U* test. *P* values less than 0.1 are displayed. *P < 0.05.

Next, we assessed if eCoV spike protein-directed FcγRIIIa-binding antibodies possibly account for the anti-SARS-CoV-2 S2 FcγRIIIa antibodies (Fig. 3 and 4). We used the antigen-specific IgG and FcγRIIIa-binding antibody ELISA to measure antibody levels against HCoV-229E spike and HCoV-OC43 spike proteins as representative antibody responses to alpha and beta eCoV infections, respectively (Fig. 5). As expected, IgG levels were higher against HCoV-229E spike (*P* = 0.0241, Fig. 5A) and HCoV-OC43 spike (*P* = 0.2086, Fig. 5B) in the individuals with a presumed recent eCoV infection, although only the comparison with HCoV-229E spike antibody levels reached statistical significance. Furthermore, HCoV-229E (*P* = 0.0688, Fig. 5C) and HCoV-OC43 (*P* = 0.0282, Fig. 5D) spike-specific FcγRIIIa-binding antibody levels were also higher in the individuals with a presumed recent eCoV infection, and these comparisons showed a statistical trend and significance. There were no statistical differences in the HCoV-229E (*P* = 0.4689, Fig. 5F) and HCoV-OC43 spike-specific (*P* = 0.2044, Fig. 5E) FcγRIIIa-binding antibodies-to-IgG ratio between those with or without a presumed recent eCoV infection.

**Fig 6:**
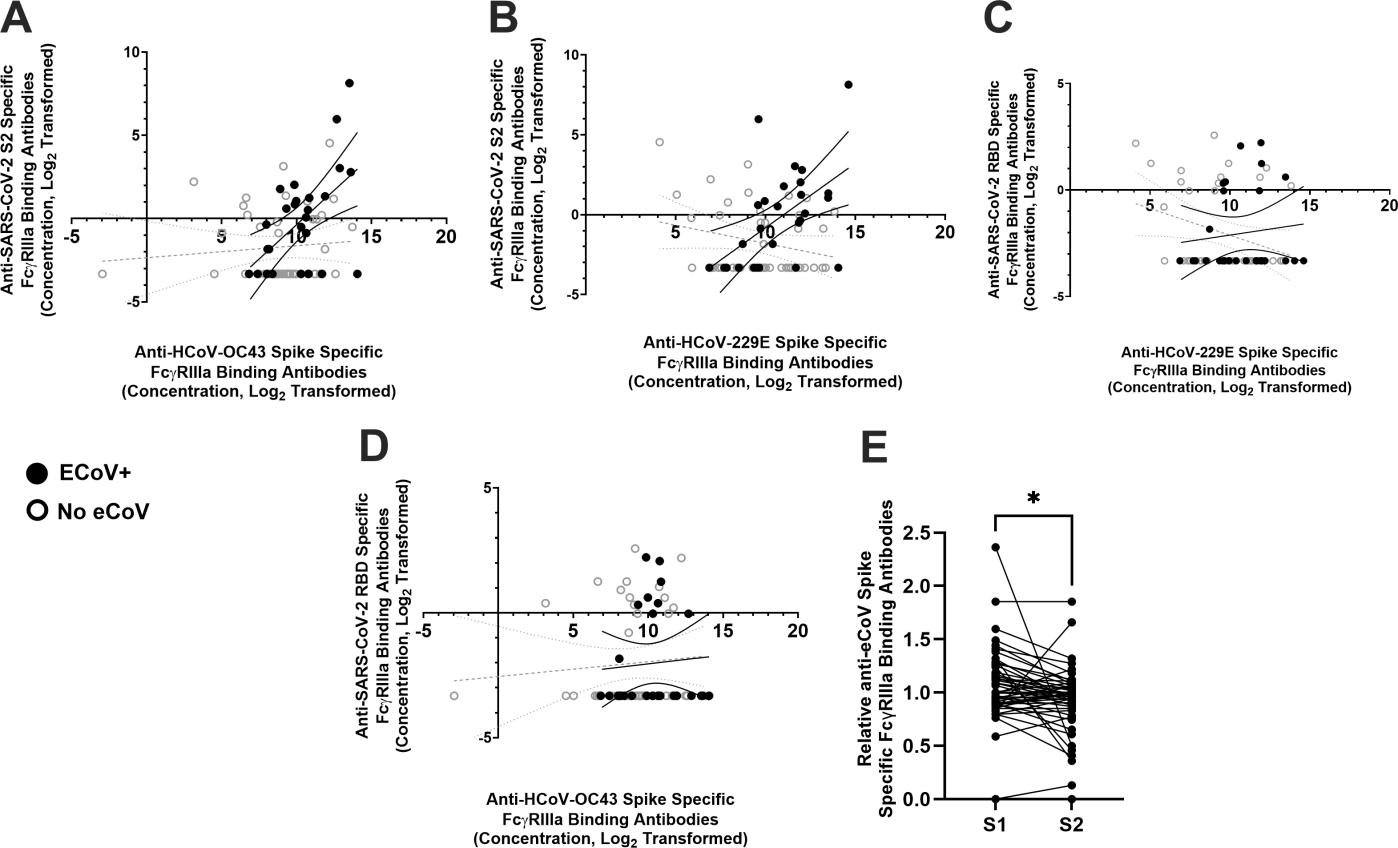
ECoV-specific antibody FcR binding responses correlate with SARS-CoV-2 S2 antibody responses. The cross-reactivity of plasma antibody FcR binding responses was measured in individuals with a presumed or documented recent eCoV infection (black circle) or without a presumed recent eCoV infection (white circle). (**A and B**) Correlations between HCoV-229E (**A**) or HCoV-OC43 (**B**) spike-specific antibody FcR binding to SARS-CoV-2 S2-specific antibody FcR binding responses. Black dots and lines represent individuals with presumed or documented recent eCoV infection, while gray represents those without a presumed recent eCoV infection. The lines show a simple linear regression with 95% CIs. (**C and D**) Correlations between HCoV-229E (**C**) or HCoV-OC43 (**D**) spike-specific antibody FcR binding to SARS-CoV-2 RBD-specific antibody FcR binding responses. (**E**) Relative HCoV-229E and HCoV-OC43 spike-specific FcR-binding antibody titers after depletion of either SARS-CoV-2 S1 or S2 spike antibodies. The SARS-CoV-2 S1 and S2 antibody-depleted samples are both normalized to antibody responses after a SARS-CoV-2 nucleocapsid depletion. Statistical analyses were performed using a Wilcoxon matched-pair signed-rank test. *P < 0.05.

We next compared the SARS-CoV-2 antigen and eCoV spike FcγRIIIa-binding antibody responses to understand the extent and specificity in cross-reactivity of the antibodies. Moderate positive correlations were observed between the SARS-CoV-2 S2 and HCoV-229E (Spearman *r* = 0.5153 and *P* = 0.010, Fig. 6A) and HCoV-OC43 (Spearman *r* = 0.4793 and *P* = 0.010, Fig. 6B) spike FcγRIIIa-binding antibody responses in those individuals with presumed or documented recent eCoV infections. There were no significant correlations in SARS-CoV-2 S2 and eCoV FcγRIIIa-binding antibody responses in individuals without a presumed recent eCoV infection (Spearman *r* = −0.1567 and *P* = 0.31, and Spearman *r* = 0.0879 and *P* = 0.57, respectively; Fig. 6A and B). Correlations were not observed between the SARS-CoV-2 RBD and HCoV-229E (Spearman *r* = 0.1099 and *P* = 0.59, Fig. 6C) and HCoV-OC43 (Spearman *r* = 0.0818 and *P* = 0.68, Fig. 6D) spike FcγRIIIa-binding antibody responses in those individuals with or without a presumed or documented recent eCoV infection.

We further elucidated the S2 specificity of the cross-reactive antibodies elicited after a recent eCoV infection by measuring eCoV FcγRIIIa-binding antibody levels after depleting SARS-CoV-2 S1 or S2 binding antibodies. We assessed the impact on a subset of individuals with presumed recent eCoV infection only because SARS-CoV-2 S2 and eCoV FcγRIIIa-binding antibodies demonstrated significant correlation (Fig. 6A and B). Change to eCoV FcγRIIIa-binding antibody levels was assessed relative to SARS-CoV-2 nucleocapsid antibody depletion, which was expected to have no effect. Depletion with SARS-CoV-2 S2 (mean 0.9339) but not S1 (mean 1.052) binding beads significantly decreased HCoV-229E and HCoV-OC43 (*P* = 0.0017, Wilcoxon matched-pair signed-rank test; Fig. 6E) spike FcγRIIIa-binding antibodies. In aggregate, these observations suggest eCoV antibodies capable of binding to FcγRIIIa are elevated after a recent eCoV infection, and these antibodies correlate with increased cross-reactivity against the SARS-CoV-2 spike S2 region.

### Recent eCoV infection does not improve T-cell responses against SARS-CoV-2 antigens

**Fig 7:**
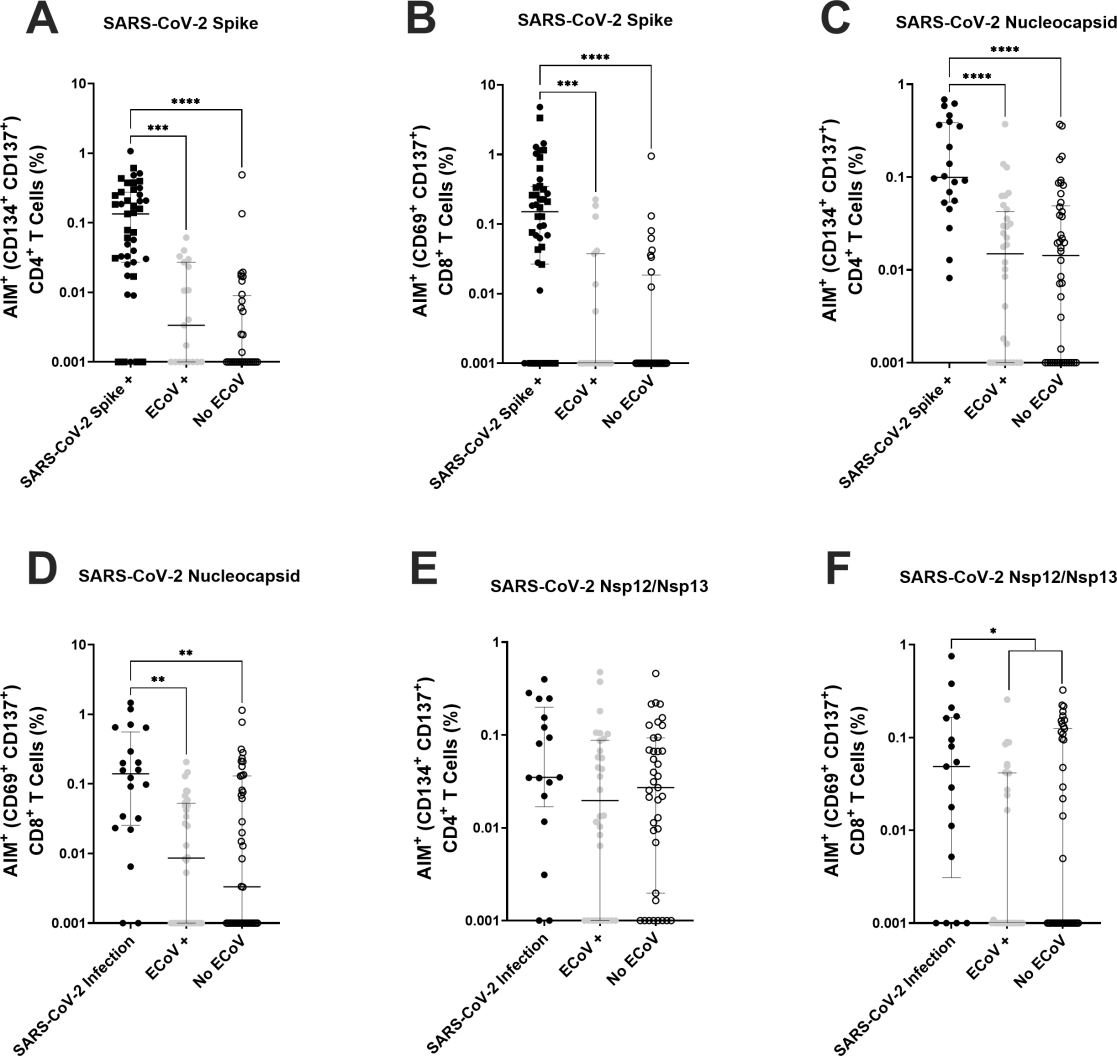
T-cell responses against SARS-CoV-2 antigens are similar between individuals with and without presumed recent eCoV infections. T-cell responses were measured in individuals with either a previous SARS-CoV-2 spike exposure (previous SARS-CoV-2 infection [black circle] and previous COVID-19 vaccination [black square]), presumed or documented recent eCoV infection (gray circle), or without a presumed recent eCoV infection (white circle). Cells were stimulated with SARS-CoV-2 peptide pools, and the percentage of activated (CD134^+^ CD137^+^) CD4^+^ T cells and (CD69^+^ CD137^+^) CD8^+^ T cells was measured. (**A and B**) T-cell activation of CD4^+^ (**A**) and CD8^+^ (**B**) was measured after stimulation with SARS-CoV-2 spike peptides. (**C and D**) T-cell activation of CD4^+^ (**C**) and CD8^+^ (**D**) was measured after stimulation with SARS-CoV-2 nucleocapsid peptides. (**E and F**) T-cell activation of CD4^+^ (**E**) and CD8^+^ (**F**) was measured after stimulation with SARS-CoV-2 nsp12/nsp13 peptides. Data were background subtracted against the negative control (dimethyl sulfoxide only). The dark horizontal lines in each scatter dot plot denote the median and interquartile range. Note that the *y *axis varies among the different panels. Statistical analyses were performed using either the Kruskal-Wallis test with Dunn multiple comparison test or the Mann-Whitney *U* test. *P* values less than 0.1 are displayed. *P < 0.05, **P < 0.01, ***P < 0.001, ****P < 0.0001.

We have previously shown that T-cell responses generated by SARS-CoV-2 infection were associated with lower incidence of eCoV infections, which demonstrated the impact of heterotypic T-cell responses among the different CoVs (4). We tested T-cell responses against SARS-CoV-2 spike, nucleocapsid, and nsp12/nsp13 antigens in the subset of individuals with available peripheral blood mononuclear cells (PBMC) and either a confirmed previous SARS-CoV-2 infection (*n* = 20), presumed or documented recent eCoV infection (*n* = 32), or no presumed recent eCoV infection (*n* = 42). The subset of individuals with available PBMCs was representative of the full cohort (Table S3). As expected, individuals with a previous SARS-CoV-2 antigen experience had significantly higher activated (CD134^+^ CD137^+^) CD4^+^ and (CD69^+^ CD137^+^) CD8^+^ T-cell responses to SARS-CoV-2 spike (Fig. 7A and B) and nucleocapsid (Fig. 7C and 5D). nsp12/nsp13 CD4^+^ (*P* = 0.1120, Fig. 7E) and CD8^+^ T-cell responses (*P* = 0.0327, Fig. 7F) were also higher in those with prior SARS-CoV-2 infection as compared to those with no previous SARS-CoV-2 antigen experience, although there were no consistent differences when compared with the two groups classified based on recent eCoV infection individually. Importantly, there were no significant differences in activated CD4^+^ or CD8^+^ T-cell responses to SARS-CoV-2 spike, nucleocapsid, or nsp12/nsp13 peptides between those individuals with or without a presumed recent eCoV infection (Fig. 7A through F). This suggests that cross-reactive T-cell levels in the peripheral blood are not significantly boosted after a recent eCoV infection.

## DISCUSSION

Multiple different highly pathogenic CoV have emerged over the past 25 years (18–20), and thus, developing a pan-CoV vaccine remains a major priority. The efficacy of this pan-CoV vaccine against future emerging CoVs may be judged by how well it performs against the currently circulating HCoVs. Such broad protection against the diverse CoVs requires knowledge of both the conserved immunodominant regions and the components of the immune system mediating this protection. Understanding the role of heterotypic immunity in the protection against SARS-CoV-2 and severe COVID-19 is a key step toward developing a pan-CoV vaccine. Most studies suggest that heterotypic immunity from a recent eCoV infection protects against severe COVID-19, while some studies indicate there is no cross-reactive protection (5, 21–24). These varying conclusions may differ based on the cohorts studied and the criteria used to define protection against SARS-CoV-2 or severe COVID-19. Mechanistic studies in different non-human models have also yielded contradictory results (25, 26). In the absence of a human challenge study, difficulty with longitudinal determination of eCoV infection in the absence of prior SARS-CoV-2 antigen exposure makes prospective natural history human studies difficult for confirming a biological mechanism. Besides resolving this controversy, it is important to understand how a recent eCoV infection affects immune responses directed against SARS-CoV-2 because it will highlight immune mechanisms that mediate cross-reactive protection. Here in this study, we identify a potential role of antibody Fc effector functions in the eCoV-derived heterotypic immune response against SARS-CoV-2.

Studies have measured heterotypic immunity against SARS-CoV-2 by correlating eCoV and SARS-CoV-2 immune responses (27, 28). In general, investigations have not identified individuals with a recent eCoV infection and assessed subsequent SARS-CoV-2 immune responses. We used both clinically documented cases and antibody levels against eCoV nucleocapsids to classify presumed recent eCoV infections. These are the most reliable and accepted ways to classify recent eCoV infection. This classification, however, is complicated by the prevalence and frequency of eCoV infections. By the age of 5, most children have had at least one eCoV infection, with reinfections occurring frequently (10, 29). The protective immunity toward these eCoVs lasts for a short duration, and reinfections can even happen within the same year despite detectable antibody levels (1). Thus, it is difficult to define a recent eCoV infection with confidence in the absence of PCR-based results or longitudinal sampling of the entire cohort. Our results and comparisons between groups largely depend on this definition of a recent eCoV infection. SARS-CoV-2-directed FcR antibodies still trended higher if the prior eCoV infection group was defined by documented infection on the CRP-PCR test within the past 550 days and up to 35% of those with the highest alpha and beta nucleocapsid IgG levels (Table S4). This sensitivity analysis provides further confidence for our conclusions. Regardless, additional studies with well-defined eCoV infections will further validate our findings.

In our study, SARS-CoV-2 S2- but not RBD-directed antibodies were elevated after a recent eCoV infection (Fig. 3). These results align with previous studies because many individuals have pre-existing anti-SARS-CoV-2 antibodies, with a preference toward more conserved regions, like S2, compared to more variable regions, like RBD (30). This S2 region can be conserved even among the diverse range of alpha and beta CoVs (31). In general, the higher degree of similarity between different CoVs predicts higher levels of antibody-mediated protection against heterologous infections (3). Sterilizing protection against a heterologous CoV requires neutralizing antibodies against conserved spike regions. In accordance with our study and previous work, eCoV-directed antibodies poorly cross-react with SARS-CoV-2 RBD and thus have limited neutralizing ability (22). Antibodies targeted against SARS-CoV-2 S2 can neutralize the virus, but they are rare and less potent than anti-SARS-CoV-2 RBD-neutralizing antibodies (30). This agrees with clinical observations that a recent eCoV infection does not reduce the number of SARS-CoV-2 infections but instead is associated with protection against COVID-19 severity (5, 21). The protection against severe COVID-19 but not SARS-CoV-2 infection indicates that components of the immune system other than neutralizing antibodies may be mediating this protection. This study identified correlates associated with the protection against severe COVID-19 in those with a recent eCoV infection. This information can be helpful for future assessment of pan-coronavirus preventative measures that are not based on neutralizing antibodies.

The Fc effector function of cross-reactive SARS-CoV-2 S2-directed antibodies could be the immune correlate that protects against severe COVID-19 (32). Multiple observations from our study support this possibility. First, SARS-CoV-2 S2 FcγRIIIa-binding antibodies were higher in those classified as having a recent eCoV infection. Second, increased SARS-CoV-2 S2 FcγRIIIa levels were associated with higher alpha eCoV nucleocapsid levels. Third, there was a direct association with eCoV spike IgG and SARS-CoV-2 S2 FcγRIIIa levels. Finally, depletion of SARS-CoV-2 S2 antibodies decreased eCoV FcγRIIIa binding. In aggregate, this implies that recent eCoV infection enhances cross-reactive SARS-CoV-2 S2 FcγRIIIa-binding antibodies. We used an ELISA that measures antigen-specific antibodies capable of binding to FcγRIIIa, and the strength of this interaction primarily depends on the fucosylation of the antibody Fc portion (17). We focused our study on FcγRIIIa, which is expressed on NK cells and macrophages, and its interaction is crucial for ADCC and ADCP activities (33). We found that both SARS-CoV-2 S2 and eCoV spike antibodies capable of binding FcγRIIIa were increased in those individuals with a recent eCoV infection. Both higher afucosylated antibody levels and greater ADCC activity are correlated with COVID-19 severity (34, 35). Yet, stronger ADCC activity is also associated with protection against fatal COVID-19 cases (16). We only measured levels and not the function of FcγRIIIa-binding antibodies, and thus, we can only speculate about increases in ADCC and ADCP. Furthermore, we restricted our analysis to peripheral blood immune responses, and cross-reactive tissue-based SARS-CoV-2 immunity may be different after recent eCoV infection.

Elevated levels of T cells early in a SARS-CoV-2 infection are associated with protection against severe COVID-19 (36, 37). These initial protective T cells against SARS-CoV-2 are speculated to be cross-reactive memory T cells from a previous eCoV infection. Although other studies have noted a boost in cross-reactive T cells after a SARS-CoV-2 infection, few studies have looked at cross-reactive T-cell levels after an eCoV infection (4, 38). Cross-reactive T cells against SARS-CoV-2 antigens were detected in nearly all SARS-CoV-2-naïve individuals, but they were at similar levels among those with or without a presumed recent eCoV infection. Our data suggests that an eCoV infection does not preferentially expand T-cell responses against SARS-CoV-2 spike, nucleocapsid, or nsp12/nsp13. Alternatively, eCoV-specific T cells have a lower avidity toward their corresponding SARS-CoV-2 peptides, so the cross-reactive T cells may not respond sufficiently in our assay (39). Furthermore, we only stimulated cells with peptides from SARS-CoV-2 spike, nucleocapsid, nsp12, and nsp13. We chose these proteins because they are either immunodominant or associated with immune protection against CoV-related diseases, but other antigens may contribute to the heterotypic immune response (4, 6, 40). Humans have a limited T-cell repertoire against SARS-CoV-2 epitopes, and thus, it is possible that the appropriate peptides were not included in our peptide pools (40). Cross-reactive T cells may still contribute to reducing COVID-19 severity, but in our cohort, they were not associated with a recent eCoV infection.

Our observations suggest that antibody Fc effector function may play a critical role in the reduction of COVID-19 severity. Antibody Fc effector functions are important in the battle against other viruses and disease, including HIV and cancer (41, 42). Most clinical versions of monoclonal antibodies are now designed to have afucosylated Fc regions, so they are more capable of mediating ADCC and ADCP activities. As work continues toward developing a pan-CoV vaccine or therapy, it will be important to understand how to elicit optimal FcR functionality.

## MATERIALS AND METHODS

### Participants and data collection

Demographic and clinical information was extracted from the BMC EMR (Table S1). A representative subset of these individuals with available PBMCs was used in the T-cell response analyses (Table S3). Pre-pandemic samples were collected from the HIV and aging cohort as previously described (43, 44). The pre-pandemic samples were collected from June 2017 to March 2020, before the first diagnosed SARS-CoV-2-infected individual at BMC. All post-pandemic samples were collected after November 2020 and prior to December 2021 before the widespread Omicron SARS-CoV-2 surge (45). The post-pandemic blood samples were obtained from BMC patients during a non-COVID-19-related medical visit. Prior to sample collection, all individuals’ status regarding COVID-19 vaccination and prior SARS-CoV-2 infection was confirmed during the consent process. Previous SARS-CoV-2 infections were based on available prior SARS-CoV-2 reverse transcription-PCR test results. Vaccinated individuals had received at least two doses of the Pfizer BioNTech BNT162b2 or Moderna mRNA-1273 COVID-19 vaccine or one dose of the Janssen/Johnson & Johnson Ad26.COV2.S COVID-19 vaccine. No individual had received a COVID-19 vaccine booster because this practice was instituted after the end of our sample collection period. Included individuals were greater than 18 years of age. Documented eCoV infections were based on prior documented positive test results for HCoV-229E, HCoV-HKU1, HCoV-NL63, or HCoV-OC43 in the CRP-PCR test. CRP-PCR tests were used to evaluate patients who presented with acute respiratory symptoms, but we did not confirm the medical reasons for the testing in all cases. The CRP-PCR was done at the discretion of the treating physician.

### SARS-CoV-2- and ECoV-specific IgG antibody quantification

SARS-CoV-2, eCoV spike, and nucleocapsid protein-specific IgG titers were detected by ELISA as previously described (28). SARS-CoV-2 RBD protein (SinoBiological, 40592-V08H), SARS-CoV-2 S2 protein (SinoBiological, 40590-V08H1), SARS-CoV-2 nucleocapsid protein (SinoBiological, 40588-V08B), HCoV-OC43 spike protein (SinoBiological, 40607-V08B), HCoV-OC43 nucleocapsid protein (SinoBiological, 40643-V07E), HCoV-229E spike protein (SinoBiological, 40605-V08B), HCoV-229E nucleocapsid protein (SinoBiological, 40640-V07E), HCoV-HKU1 nucleocapsid protein (SinoBiological, 40642-V07E), or HCoV-NL63 nucleocapsid protein (SinoBiological, 40641-V07E) was used in these ELISAs. After overnight incubation with an antigen, wells were blocked with casein blocking buffer (Thermo Fisher Scientific, 37528). Three plasma dilutions were tested (1:5, 1:100, and 1:2,000), and IgG was detected using anti-human horseradish peroxidase (HRP)-conjugated secondary antibodies for IgG detection (diluted 1:50,000; Sigma-Aldrich, A0170) with 3,3′,5,5′ tetramethylbenzidine-ELISA substrate solution (Thermo Fisher Scientific, 34029). The reaction was stopped using 2 M sulfuric acid, and absorbance was measured on a SpectraMax190 Microplate Reader (Molecular Devices) at 450 nm. The optical density from the no-antigen negative control wells was subtracted from all readings. A positive control standard (CR3022 IgG; Abcam, 273073) was serially diluted and measured against SARS-CoV-2 RBD to create a standard curve on each plate. The CR3022 standard curve was used to calculate titers (relative units) for each sample by interpolating a four-parameter logistic curve.

### Pseudovirus neutralization assay

The VSV-ΔG based pseudoviruses expressing SARS-CoV-2-Wuhan spikes were produced by transfecting SARS-CoV-2-spike protein (BEI Resources, NR52310) expression plasmids, infecting with VSV-G pseudotyped virus (G*ΔG-VSV), and collecting supernatants previously described (4). Pseudovirus neutralization assays were conducted as previously described (46). Briefly, heat-inactivated plasma was serially diluted using a twofold serial dilution series starting at a 1:40 dilution and incubated with 1.25 × 10^4^ Vero E6 cells. Luciferase expression was measured using the Promega Bright-Glo Luciferase Assay System (Thermo Scientific) on a SpectraMax190 Microplate Reader (Molecular Devices). Percent neutralization was calculated in comparison to luciferase expression in infected wells without patient plasma. Area under the curve values were calculated from the curve generated from the neutralizations across the serially diluted plasma (47). All neutralizations were tested in triplicate for a minimum of two independent times. Neutralization against VSV-G pseudotyped virus (G*ΔG-VSV) was used as a control to assess activity against VSV-G protein in the absence of a CoV spike protein.

### SARS-CoV-2- and ECoV-specific antibody FcR binding quantification

Levels of SARS-CoV-2 and eCoV spike and nucleocapsid protein-specific antibodies with the ability to bind the Fc receptor, FcγRIIIa, were detected by the fucose-sensitive ELISA-based method of antigen-specific IgG Fc fucosylation (17). The ELISA protocol for antigen-specific IgG quantification, as described above, was adapted to include measurements of antibody binding to FcγRIIIa. The following additions were made to the previously described ELISA protocol. Plasma was diluted 1:5 to 1:50. Either 1 μg/mL of His-tagged (Invitrogen, RP-87975) or biotinylated (gift from Dr. Gestur Vidarsson, PhD) FcγRIIIa was used as FcR antigens. Antibody-bound FcR was detected by either anti-His HRP-conjugated antibody (1 μg/mL; Invitrogen, MA1-21315-HRP) or HRP-conjugated streptavidin (0.125 μg/mL; Thermo Scientific, N100). Similar methods of developing, reading, and calculating values for the ELISA were used as described earlier.

### SARS-CoV-2 antigen-specific antibody plasma depletion

Streptavidin magnetic beads (Thermo Scientific, 88816) were coated with biotinylated SARS-CoV-2 antigens: S1 (SinoBiological, 40591-V08H-B), S2 (SinoBiological, 40590-V08B-B), or nucleocapsid (SinoBiological, 40588-V08B-B). The bead-protein complexes were then incubated with plasma at room temperature. The bound bead-protein-antibody complexes were removed from the plasma using a magnet. The antibody-depleted plasma samples were used in previously described ELISAs. The responses after SARS-CoV-2 S1- and S2-specific antibody depletions were normalized relative to the spike-specific antibody responses after SARS-CoV-2 nucleocapsid antibody depletion.

### SARS-CoV-2-specific T-cell responses

The activation-induced marker assay to measure antigen-specific T-cell responses was performed as described previously (48, 49). SARS-CoV-2 spike protein (BEI Resources, NR-52402), SARS-CoV-2 nucleocapsid (BEI Resources, NR-52404), SARS-CoV-2 nsp12 protein (JPT, PM-WCPV-NSP12-2), and SARS-CoV-2 nsp13 protein (JPT, PM-WCPV-NSP13-2) pools were added at a final concentration of 1 µg/mL (containing less than 0.1% dimethyl sulfoxide [DMSO]) for the stimulation. Media with 0.1% DMSO were used as a negative control.

After stimulation, the cells were fixed using cold BD Cytofix Fixation Buffer (1:10 dilution; BD Biosciences, 554655) and blocked using human Fc receptor block (1:100 dilution; BioLegend, 422302). Live cells were then stained for T-cell lineage and activation markers: LIVE/DEAD cell marker (1:200 dilution; Thermo Fisher, L23105), Alexa Fluor 647 anti-human CD3 (1:100 dilution; BioLegend, clone HIT3a, 300321), Alexa Fluor 488 anti-human CD4 (1:200 dilution; BioLegend, clone SK3, 344618), allophycocyanin/Fire 750 anti-human CD8a (1:50 dilution; BioLegend, clone HIT8a, 300931), phycoerythrin (PE)/Cyanine7 anti-human CD69 (1:50 dilution; BioLegend, clone FN50, 310911), Brilliant Violet 421 anti-human CD134 (OX40) (1:25 dilution; BioLegend, clone Ber-ACT35 [ACT-35], 350013), and PE anti-human CD137 (4–1BB) (1:100 dilution; BioLegend, clone 4B4-1, 309803). Stained cells were analyzed on either a BD LSR II Flow Cytometer (BD Biosciences) or Cytek Aurora 5L (Cytek Biosciences).

The resulting flow cytometry data were analyzed using FlowJo software using similar methods as previously described (4). Live (LIVE/DEAD marker^−^) T cells (CD3^+^) were then gated on either CD4^+^ or CD8^+^. Activated CD4^+^ was defined by the double-positive CD134^+^ CD137^+^ population, while activated CD8^+^ was defined by the double-positive CD69^+^ CD137^+^ population. The percentage of activated T cells for a given antigen stimulation condition was then background subtracted against the negative control (DMSO only) condition.

### Statistical analysis

Individuals with a previous documented SARS-CoV-2 infection or previous COVID-19 vaccination were grouped together as previous SARS-CoV-2 spike exposure for all spike-directed immune response assessments. Individuals with a previous COVID-19 vaccination were grouped based on their classified eCoV infection status for the non-spike directed immune response examinations. Composite alpha or beta eCoV nucleocapsid antibody scores were created by log2 transforming the average of HCoV-229E and HCoV-NL63 or HCoV-HKU1 and HCoV-OC43 IgG titers, respectively. Individuals with either a documented eCoV infection in the past 550 days or those within the top 25% with the highest alpha or beta eCoV nucleocapsid antibody scores were classified as presumed or documented eCoV infection. The remaining SARS-CoV-2 infection-naïve individuals were considered without a recent eCoV infection. Comparison of responses in individuals with a previous SARS-CoV-2 infection or SARS-CoV-2 spike exposure to the SARS-CoV-2 infection-naïve groups was conducted using Kruskal-Wallis tests and corrected with the Dunn multiple comparison test. Mann-Whitney *U* tests were used for direct comparisons between individuals with or without a presumed recent eCoV infection. Wilcoxon matched-pair signed-rank tests were used for direct comparisons between matched samples after different antibody depletions. Correlations were assessed using Pearson and Spearman tests as appropriate. Categorical differences were examined using the Fisher exact test or chi-square test when comparing more than two groups. In the multivariable logistic regression model, the presence or absence of a SARS-CoV-2 antibody binding FcγRIIIa response was the dependent variable. Alpha and beta eCoV nucleocapsid IgG levels, prior SARS-CoV-2 spike experience (categorical variable), and available demographic factors and comorbidities were independent predictors in the multivariate analysis. All covariates in a univariate model with *P* values less than or equal to the 0.15 level were initially included in the multivariable model. Covariates with *P* values greater than 0.10 in the multivariable model were then removed. Statistical analyses were performed using GraphPad Prism version 9.0.2. A two-sided *P* value less than 0.05 was considered statistically significant.

## Data Availability

All data are available in the article and supplemental material or from the corresponding author upon request.

## SUPPLEMENTAL MATERIAL

Supplemental material jvi.00550-25-s0001.docx Figures S1 and S2; Tables S1 to S4.

ASM does not own the copyrights to Supplemental Material that may be linked to, or accessed through, an article. The authors have granted ASM a non-exclusive, world-wide license to publish the Supplemental Material files. Please contact the corresponding author directly for reuse.
