# Supplementary material for: Endemic coronavirus infection is associated with SARS-CoV-2 Fc receptor-binding antibodies
Source: J Virol. 2025 May 19;99(6):e00550-25. doi: 10.1128/jvi.00550-25 (PMC12172460; doi:10.1128/jvi.00550-25)
Supplement: Supplemental material — Figures S1 and S2; Tables S1 to S4. [file jvi.00550-25-s0001.docx]

**
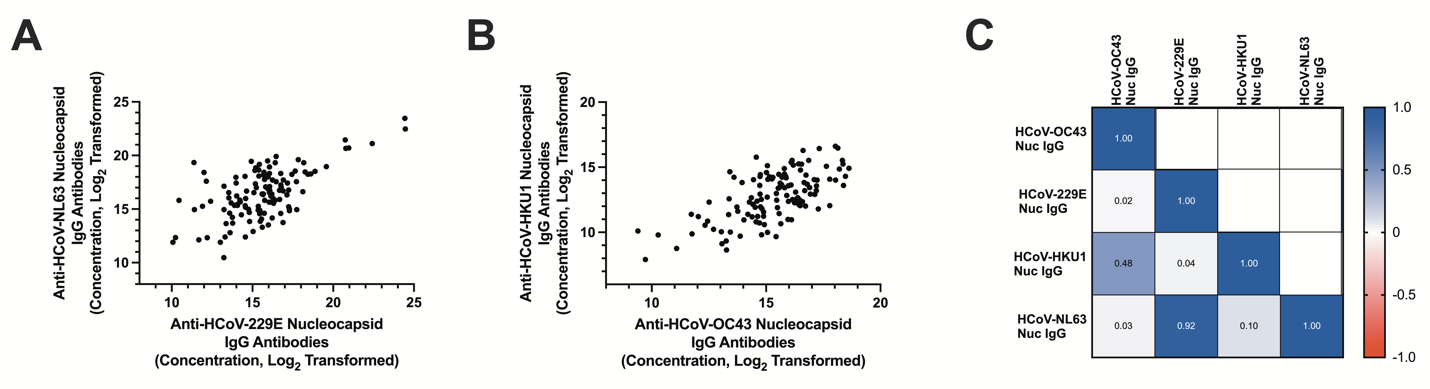
**

**Figure S1. Correlations between IgG antibody responses to alpha and beta eCoV nucleocapsid proteins.** Anti-nucleocapsid antibody responses were measured in all individuals in the study. (A) Correlation of IgG antibody responses against HCoV-229E and HCoV-NL63 nucleocapsids. (B) Correlation of IgG antibody responses against HCoV-HKU1 and HCoV-OC43 nucleocapsids. (C) The matrix shows Pearson correlation r values between IgG antibody responses against HCoV-OC43, HCoV-229E, HCoV-HKU1, and HCoV-NL63 nucleocapsid proteins. Blue represents stronger positive correlations, while red signifies stronger negative correlations.

**
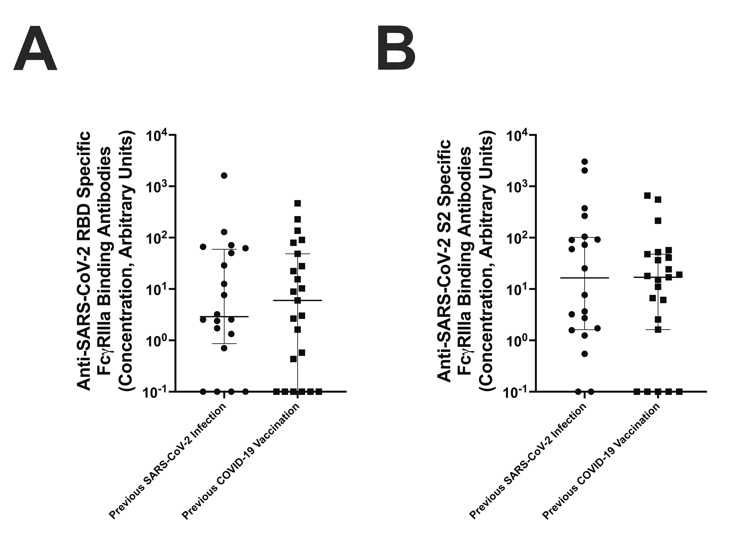
**

**Figure S2. SARS-CoV-2 spike specific Fc receptor binding antibody responses.** FcR binding antibody responses in those with documented previous SARS-CoV-2 infection (black circle) or prior COVID-19 vaccination (black square). (A and B) Titer of SARS-CoV-2 RBD (A) or SARS-CoV-2 S2 (B) specific antibodies binding to the Fc receptor, FcγRIIIa. The dark horizontal lines in each scatter dot plot denote the median and interquartile range. Statistical analyses were performed using the Mann-Whitney U test.

Table S1. Demographics of the individuals with collected blood specimens for analyses of SARS-CoV-2 and eCoV mediated antibody responses

|  | **Prior**  **SARS-CoV-2 infection /**  **No COVID-19 vaccine**  **(n = 20)** | **Prior**  **COVID-19 vaccine /**  **No**  **SARS-CoV-2 infection**  **(n = 29)** | **No prior**  **SARS-CoV-2 infection or**  **COVID-19 vaccine**  **(n = 78)** | **p-value ^a^** |
| --- | --- | --- | --- | --- |
| **Age (years),**  **median (IQR)** | 53 (48 – 59) | 62 (54 – 70) | 56 (46 – 64) | 0.0215 **^b^** |
| **Male** | 8 (40) | 19 (66) | 36 (50) | 0.1834 |
| **Race/Ethnicity ^c^** | | | | 0.3087 |
| **Black** | 14 (70) | 13 (45) | 35 (49) |  |
| **White** | 3 (15) | 13 (45) | 26 (36) |  |
| **Hispanic/Latino** | 3 (15) | 3 (10) | 8 (11) |  |
| **Other/missing** | 0 (0) | 0 (0) | 3 (4) |  |
| **Diabetes mellitus** | 5 (25) | 10 (34) | 21 (29) | 0.7640 |
| **Heart disease ^d^** | 3 (15) | 12 (41) | 15 (21) | 0.0520 |
| **Lung disease ^e^** | 5 (25) | 8 (28) | 26 (36) | 0.5322 |
| **CKD ^f^** | 1 (5) | 3 (10) | 6 (8) | 0.7997 |
| **HIV ^g^** | 7 (35) | 5 (17) | 26 (36) | 0.1686 |
| **Cancer** | 1 (5) | 2 (7) | 1 (1) | 0.3367 |
| **Number of co-morbidities ^h^** |  | | | 0.6309 |
| **0** | 3 (15) | 5 (17) | 12 (17) |  |
| **1** | 12 (60) | 12 (41) | 33 (46) |  |
| **≥2** | 5 (25) | 12 (41) | 17 (38) |  |
| **Pre-Pandemic ^i^** | - | - | 14 (26) | **^-^** |

Data shows number and percent unless otherwise indicated, ^a^ Chi-square test unless otherwise indicated, ^b^ Kruskal-Wallis test and Dunn’s multiple comparison test, ^c^ As specified in the EMR; an individual may be in more than 1 category, ^d^ Heart disease includes coronary artery disease and congestive heart failure, ^e^ Lung disease includes chronic obstructive pulmonary disease and asthma, ^f^ Chronic kidney disease, ^g^ Human immunodeficiency virus, ^h^ Number of comorbidities accounts for diabetes mellitus, heart disease, lung disease, chronic kidney disease, HIV, and cancer, ^i^ Samples collected before March 2020

Table S2. The accuracy of SARS-CoV-2 exposure classification based on the established cutoffs for classifying individuals into the specified groups.

|  |  |  | **Clinical Classification** | | |  | |
| --- | --- | --- | --- | --- | --- | --- | --- |
|  |  |  | **Prior**  **SARS-CoV-2 infection**  **(n = 20)** | **COVID-19 vaccination, but no**  **SARS-CoV-2 infection**  **(n = 29)** | **Pre-pandemic**  **(n = 18)** | **Positive predictive value** | **Negative predicative value** |
| **Cutoff Classification** | **Prior infection** | RBD IgG ≥ 7.95 | 18 | 6 | 0 | 75%  (53%, 90%) | 95%  (84%, 99%) |
|  | **Vaccination,**  **no infection** | RBD IgG ≥ 7.95 & NC IgG ≤ 12.2 | 0 | 22 | 0 | 100%  (85%, 100%) | 84%  (71%, 94%) |
|  | **No**  **SARS-CoV-2 exposure** | RBD IgG < 7.95 | 2 | 1 | 18 | 86%  (64%, 97%) | 100%  (92%, 100%) |
|  | **Sensitivity** |  | 90%  (68%, 99%) | 76%  (56%, 90%) | 100%  (81%, 100%) | **Overall Accuracy 87%**  **(76%, 94%)** | |
|  | **Specificity** |  | 87%  (74%, 95%) | 100%  (91%, 100%) | 94%  (83%, 99%) |  |  |

The values in parentheses display the 95% CI.

Table S3. Demographics of the individuals with available PBMCs for analyses of eCoV mediated cellular responses against SARS-CoV-2 antigens

|  | **Prior**  **SARS-CoV-2 infection /**  **No COVID-19 vaccine**  **(n = 20)** | **Presumed or documented recent eCoV infection**  **(n = 32)** | **No presumed recent eCoV infection**  **(n = 42)** | **p-value ^a^** |
| --- | --- | --- | --- | --- |
| **Age (years),**  **median (IQR)** | 53 (48 – 59) | 65 (57 – 71) | 57 (44 – 62) | 0.0057 **^b^** |
| **Male** | 8 (40) | 17 (53) | 22 (52) | 0.6004 |
| **Race/Ethnicity ^c^** | | | | 0.3752 |
| **Black** | 14 (70) | 12 (38) | 21 (50) |  |
| **White** | 3 (15) | 14 (44) | 15 (36) |  |
| **Hispanic/Latino** | 3 (15) | 5 (16) | 5 (12) |  |
| **Other/missing** | 0 (0) | 1 (3) | 1 (2) |  |
| **Diabetes mellitus** | 5 (25) | 12 (38) | 15 (36) | 0.6216 |
| **Heart disease ^d^** | 3 (15) | 13 (41) | 9 (21) | 0.0751 |
| **Lung disease ^e^** | 5 (25) | 13 (41) | 8 (19) | 0.1156 |
| **CKD ^f^** | 1 (5) | 2 (6) | 5 (12) | 0.5633 |
| **HIV ^g^** | 7 (35) | 8 (25) | 14 (33) | 0.6716 |
| **Cancer** | 1 (5) | 1 (3) | 1 (2) | 0.8601 |
| **Number of co-morbidities ^h^** |  | | | 0.3873 |
| **0** | 3 (15) | 5 (16) | 8 (19) |  |
| **1** | 12 (60) | 11 (34) | 19 (45) |  |
| **≥2** | 5 (25) | 16 (50) | 15 (36) |  |
| **Pre-Pandemic ^i^** | - | 4 (13) | 14 (33) | 0.0553 **^j^** |
| **COVID-19 Vaccine** | - | 13 (41) | 10 (24) | 0.1368 **^j^** |

Data shows number and percent unless otherwise indicated, ^a^ Chi-square test unless otherwise indicated, ^b^ Kruskal-Wallis test and Dunn’s multiple comparison test, ^c^ As specified in the EMR; an individual may be in more than 1 category, ^d^ Heart disease includes coronary artery disease and congestive heart failure, ^e^ Lung disease includes chronic obstructive pulmonary disease and asthma, ^f^ Chronic kidney disease, ^g^ Human immunodeficiency virus, ^h^ Number of comorbidities accounts for diabetes mellitus, heart disease, lung disease, chronic kidney disease, HIV, and cancer, ^i^ Samples collected before March 2020, ^j^ Fisher’s Exact Test

Table S4. Statistical comparison of SARS-CoV-2 S2-specific antibody FcγRIIIa binding responses between individuals with or without a presumed recent eCoV infection

| Percent of individuals with highest anti-alpha and beta eCoV nucleocapsids IgG levels  included in the presumed recent eCoV group | Presumed or documented recent eCoV infection (median, IQR) | No presumed recent eCoV infection (median, IQR) | p-value ^a^ |
| --- | --- | --- | --- |
| 25% | 1.662 (0.587-3.165) | 0.100 (0.100-0.977) | 0.0234 |
| 30% | 1.436 (0.280-2.369) | 0.100 (0.100-0.977) | 0.0629 |
| 35% | 1.071 (0.145-2.298) | 0.100 (0.100-1.021) | 0.1037 |

^a^ Mann-Whitney test between groups with or without a presumed recent eCoV infection
